# From lifestyle psychiatry to whole person health: Evidence, dissemination, and scalable approaches

**DOI:** 10.1192/j.eurpsy.2025.10133

**Published:** 2025-11-18

**Authors:** Andrea Fiorillo, Joseph Firth, Blazej Misiak, Martina Rojnic Kuzman, Jerzy Samochowiec, Gaia Sampogna, Ivona Šimunović Filipčić, Dan Siskind, Simavi Vahip, Davy Vancampfort

**Affiliations:** 1 Department of Psychiatry, University of Campania “Luigi Vanvitelli”, Naples, Italy; 2 Division of Psychology and Mental Health, Manchester Academic Health Science Centre, University of Manchester, Manchester, UK; 3 Greater Manchester Mental Health NHS Foundation Trust, Manchester Academic Health Science Centre, Manchester, UK; 4 Department of Psychiatry, Wroclaw Medical University, Wroclaw, Poland; 5 Department of Psychiatry and Psychological Medicine, University Hospital Center Zagreb, Zagreb, Croatia; 6 School of Medicine, University of Zagreb, Zagreb, Croatia; 7 Department of Psychiatry, Pomeranian Medical University, Szczecin, Poland; 8 Faculty of Dental Medicine and Health, Josip Juraj Strossmayer University of Osijek, Osijek, Croatia; 9 Faculty of Health, Behavioural Science, and Medicine, University of Queensland, Brisbane, Australia; 10 Metro South Addiction and Mental Health Service, Metro South Health, Brisbane, QLD, Australia; 11 Physical and Mental Health Stream, Queensland Centre for Mental Health Research, Brisbane, Australia; 12 Affective Disorders Unit, Department of Psychiatry, Ege University Medicine Faculty, Izmir, Turkey; 13 Department of Rehabilitation Sciences, KU Leuven, Leuven, Belgium; 14 University Psychiatric Centre KU Leuven, Leuven-Kortenberg, Belgium; 15 KU Leuven Brain Institute, Leuven, Belgium

**Keywords:** lifestyle, physical health, premature mortality, severe mental disorders

## Abstract

People with severe mental illness have a life expectancy approximately 15–20 years shorter than the general population. Research is focusing on the identification of the biological and psychosocial factors contributing to this premature mortality. The need to focus on the interplay between physical and mental health has been repeatedly stated, but at the moment, a few clinical strategies have been implemented worldwide. The European Psychiatric Association has recently launched six task forces, each of them dealing with the critical areas of mental health practice. The task force on “Whole Person Health” aims to promote the integration of mental and physical health through lifestyle-related practices and address multimorbidity and premature mortality among people with severe mental illness through a series of educational, clinical, advocacy levels activities, which are briefly outlined in this paper.

## Introduction

People with severe mental illness have a life expectancy approximately 15 to 20 years shorter than the general population [1]. In recent years, there has been growing clinical and research interest in identifying determinants of this premature mortality [2] and developing strategies to improve both life expectancy and quality of life, [3] across the full spectrum of mental illnesses, highlighting premature mortality as a transdiagnostic challenge. Potential contributors can be classified as distal (i.e., contextual, system-level) or proximal (i.e., individual-level). Distal factors, including historical separation of psychiatry from other medical disciplines [4], persistent mind–body dualism, and the pervasive stigma and discrimination faced by people with mental disorders, are not the primary focus of this viewpoint, but do create the underlying environment for the propagation and poor outcomes of the “proximal factors”, discussed herein. The most important proximal factors include increased rates of adverse health behaviors (e.g., tobacco usage, sedentary behavior, poor diet), risk of co-occurring cardiometabolic diseases and other physical illnesses (partly resulting from medication side-effects), and decreased individual usage of physical healthcare (linked to inaccessibility/unavailability, diagnostic overshadowing, and other distal factors) [5]. In response, several initiatives have been launched worldwide to promote healthier lifestyles, reduce the burden of physical comorbidities and multimorbidity, and enhance awareness of overall health beyond symptom control. The European Psychiatric Association, within its Presidential Action Plan 2025–2027 [6], has recently launched a series of task forces to address some of the most pressing gaps in psychiatric practice. The six task forces will focus on: (a) treatment delivery and new care models; (b) precision psychiatry (refined diagnostic and therapeutic approaches); (c) brain and mental health across the lifespan; (d) lifestyle, multimorbidity and premature mortality; (e) public mental health (prevention of mental disorders and promotion of mental health); and (f) protection of patients’ rights and minority mental health.

## The EPA Whole Person Health taskforce

The task force on lifestyle and multimorbidity (now renamed “Whole Person Health”) has set an agenda for the coming years that includes: (a) promoting integration of mental and physical health through lifestyle-related practices, and (b) addressing multimorbidity and premature mortality among people with severe mental illness.

Specifically, the task force seeks to:

1. Strengthen collaboration with other medical specialties, including, i.e., oncology, cardiology, and endocrinology;
2. Raise awareness on the importance of lifestyle behaviors and the physical health of people with mental disorders;
3. Improving training of psychiatric professionals and medical doctors in lifestyle interventions and physical health monitoring, and clinical management of comorbidities and multimorbidity;
4. Narrow the persistent implementation gap between evidence and practice.

While there is robust evidence supporting the first three aims, implementation of good clinical practice to improve patients’ physical health remains poor in real-world psychiatric care [7]. Bridging this gap requires more than generating evidence. It calls for scalable strategies to enhance the dissemination of guidelines, system-level incentives to support the adoption of clinical recommendations, and practical toolkits to facilitate the implementation of lifestyle-based interventions across diverse mental health settings. Interventions should target the general population, people with mental disorders, physicians, and mental health professionals.

For the general population, primary prevention initiatives should prioritize: (a) health literacy on physical and mental health; (b) awareness about the importance of coping mechanisms and healthy lifestyle behaviors, such as a healthy diet, regular moderate intensity physical activity, avoiding smoking and excessive alcohol use, and sleep hygiene; and (c) promoting community initiatives that enable early detection and timely care.

For people with mental disorders, the above general population primary prevention initiative should be augmented by strategies that include: (a) psychosocial interventions to foster sustainable lifestyle changes [8–10]; (b) systematic monitoring of metabolic and cardiovascular side-effects of psychotropic medications [11]; (c) closer collaboration between mental health providers, general practitioners and other specialists; (d) anti-stigma initiatives to ensure equitable access to high quality care.

Educational resources and clinical guidance papers on lifestyle behaviors and physical health in people with mental disorders should be developed and disseminated among medical students, psychiatric residents, clinical psychiatrists, and other health professionals. Interdisciplinary collaboration should be embedded at all levels, that is, clinical, educational, and policy, rather than remaining aspirational.

The EPA Task Force aims to deliver on these objectives by:

1. Improving and strengthening partnerships with European associations in cardiology, endocrinology, oncology, diabetes, physical disability, and general medicine;
2. Developing guidance documents and practical toolkits on the implementation of lifestyle interventions and on the reduction of multimorbidity;
3. Sharing evidence-based resources with European mental health professionals;
4. Preparing accessible lifestyle toolkits for the general population;
5. Promoting a culture of health within psychiatry that is both actionable and scalable across all European countries.

These efforts will require coordination with governments, policymakers, national psychiatric associations, and European leadership to move beyond rhetoric into practice.

## Five priority areas for action

The areas with the greatest unmet need include nutrition, physical activity, sleep, substance use, social connectedness, and stress management (Table 1).

1. Nutrition: People with severe mental disorders often have poor diet characterized by high-fat and processed food intake, and low consumption of water, fresh fruit, vegetables, fish, or unprocessed meats. This leads to obesity and cardiovascular diseases [5]. Nutritional support must be extended to both patients and families, emphasizing its dual impact on physical and mental health [8, 12]. Within this, coaching in the practical sides of maintaining a healthy diet (i.e., cooking nutritious meals, affordable shopping for nutritious foods, etc.) should be emphasized over the education of physiological/biological effects of dietary nutrients.
2. Physical activity: Evidence consistently shows that moderate intensity physical activity improves both mental and physical health outcomes, and might reduce social isolation, improve cognition, and alleviate social anxiety, depressive, and psychotic symptoms [9, 10, 13]. While some major psychiatric institutions in Europe have introduced structured programs, implementation remains fragmented and poorly integrated into routine care [10]. Policy-level incentives are needed to move beyond tokenistic recommendations, to provide evidence-based exercise interventions as standard in/through mental healthcare services.
3. Sleep: Sleep symptoms are nearly universal in mental disorders, and they can also represent a stand-alone syndrome. Integrating the identification and appropriate management of sleep symptoms into psychiatric care and educational curricula of medical doctors and all mental health professionals is essential, yet remains undervalued [8, 14].
4. Substance use: Co-morbidity of substance use and mental disorders is highly prevalent, yet services often remain siloed. With rising alcohol and novel drug use among youth, comprehensive prevention campaigns starting in schools, alongside integrated dual-diagnosis services, are urgently needed [15].
5. Social connectedness and stress: The COVID-19 pandemic [16] exacerbated loneliness and stress, particularly in vulnerable populations [17]. While the harms of excessive online engagement are well documented, the protective effects of social cohesion remain underexplored [18]. Strengthening social networks and stress-management initiatives should be recognized as core elements of mental health promotion, not optional add-ons [19].

**Table 1.** Five areas of urgent action

Nutrition	Poor diet linked to obesity, cardiovascular diseases, and depression
Physical activity	Protective for heart diseases, diabetes, depression, anxiety, and cognitive decline
Sleep	Short/poor sleep is associated with metabolic dysfunction and mood disorders
Substance use	Smoking, alcohol, and drug abuse increase the risk for both physical and mental disorders
Social connectedness and stress management	Loneliness and chronic stress are well-known risk factors for cognitive decline, depression, and cardiovascular diseases

## Conclusion

The aims of the “Whole Person Health” task force align with the “One Health” paradigm, emphasizing integration of services across physical, mental, and social health domains. Achieving this vision requires moving beyond aspirational statements to enforceable actions that embed lifestyle and physical health monitoring into psychiatric practice. Ultimately, psychiatrists and mental health professionals should not limit themselves to treating solely the psychiatric symptoms of mental illness. We must embrace whole-person care, recognizing patients as individuals with intertwined physical, psychological, and social needs [20]. This is what our patients consistently request for, and it should be the defining mission of modern psychiatry.
